# Quantitative detection of *ALK* fusion breakpoints in plasma cell-free DNA from patients with non-small cell lung cancer using PCR-based target sequencing with a tiling primer set and two-step mapping/alignment

**DOI:** 10.1371/journal.pone.0222233

**Published:** 2019-09-12

**Authors:** Kei Kunimasa, Kikuya Kato, Fumio Imamura, Yoji Kukita

**Affiliations:** 1 Department of Thoracic Oncology, Osaka International Cancer Institute, Osaka, Osaka, Japan; 2 Laboratory of Medical Genomics, Nara Institute of Science and Technology, Ikoma, Nara, Japan; Iwate Medical University School of Medicine, JAPAN

## Abstract

**Background:**

Tyrosine kinase inhibitors targeted to anaplastic lymphoma kinase (ALK) have been demonstrated to be effective for lung cancer patients with an *ALK* fusion gene. Application of liquid biopsy, i.e., detection and quantitation of the fusion product in plasma cell-free DNA (cfDNA), could improve clinical practice. To detect *ALK* fusions, because fusion breakpoints occur somewhere in intron 19 of the *ALK* gene, sequencing of the entire intron is required to locate breakpoints.

**Results:**

We constructed a target sequencing system using an adapter and a set of primers that cover the entire *ALK* intron 19. This system can amplify fragments, including breakpoints, regardless of fusion partners. The data analysis pipeline firstly detected fusions by alignment to selected target sequences, and then quantitated the fusion alleles aligning to the identified breakpoint sequences. Performance was validated using 20 cfDNA samples from *ALK*-positive non-small cell lung cancer patients and samples from 10 healthy volunteers. Sensitivity and specificity were 50 and 100%, respectively.

**Conclusions:**

We demonstrated that PCR-based target sequencing using a tiling primer set and two-step mapping/alignment quantitatively detected *ALK* fusions in cfDNA from lung cancer patients. The system offers an alternative to existing approaches based on hybridization capture.

## Introduction

Anaplastic lymphoma kinase (*ALK*) gene fusions are found in 3–7% of non-small cell lung cancer (NSCLC) patients [1, 2]. The expected protein structures translated from fused *ALK* transcripts have an ALK kinase domain at the 3’ region and a fusion partner’s coiled-coil domain at the 5’ terminus [3, 4]. *ALK* fusions are considered to induce constitutive active dimeric forms using these domains in the absence of ligands, whereas normal ALK proteins bind their ligands and form activated dimers.

Tyrosine kinase inhibitors (TKIs), such as crizotinib and other next generation ALK inhibitors, have demonstrated efficacy for *ALK* fusion-positive patients in many clinical studies [1]. There is a need for molecular diagnostic tools to identify the status of *ALK*, such as gene amplification and fusion, in NSCLC patients. For diagnosis of *ALK* fusions, immunohistochemistry (IHC) and fluorescence *in situ* hybridization (FISH) of biopsy and/or surgical sections are commonly used in current clinical practice. Alternatively, reverse transcription PCR (RT-PCR) of extracted RNA is a simple method to detect known fusion types, such as echinoderm microtubule associated protein-like 4 (*EML4*)-*ALK* variants [5, 6].

Most *ALK* fusions are caused by genomic rearrangement involving intron 19 of *ALK* and an intron of a partner gene. In particular, *EML4*-*ALK* fusions that are commonly observed in NSCLC are the results of inversions of the short arm of chromosome 2, where both genes are located [3]. Because genomic fusion breakpoints do not occur at fixed positions, assignment of breakpoints requires sequencing of the entire intronic region.

“Liquid biopsy” is an emerging technology which uses plasma cell-free DNA (cfDNA) instead of DNA from tumor tissue for diagnostic purposes in the field of oncology. cfDNA is almost randomly fragmented to approximately 170 bp, and cfDNA from cancer patients includes circulating tumor DNA (ctDNA) derived from dying/dead tumor cells/tissues in cancer patients. Because the level of ctDNA correlates with disease progression, quantitation of ctDNA is clinically important. Several groups have developed next-generation sequencing (NGS)-based technologies to target exons of driver genes and/or mutation hotspots for diagnosis of cancer patients [7–10]. Detection of *ALK* fusions in cfDNA have been performed using hybridization-capture-based target sequencing on the Illumina platform [9, 11, 12]. In these assays, whole cfDNA fragments attached to adapters are amplified, and the targets (*ALK* fusions) captured using tiling oligonucleotide hybridization probes homologous to *ALK* intron 19, and then enriched with bead technologies. Concentrated target regions can then be subjected to library construction and DNA sequencing. However, the assay process may introduce inaccurate quantitation because whole genome amplification methods are known to cause amplification biases [13]. In this study, we constructed a target sequencing system using an adapter and a set of primers that cover the entire region of *ALK* intron 19. This system directly amplifies regions including breakpoints, regardless of fusion partners, and without using hybridization capture. We also constructed an analysis pipeline for quantitative detection of *ALK* fusions. The performance of the system was validated using cfDNA from *ALK*-positive NSCLC patients and healthy volunteers.

## Results

### Development of the ALK fusion detection pipeline

**Fig 1:**
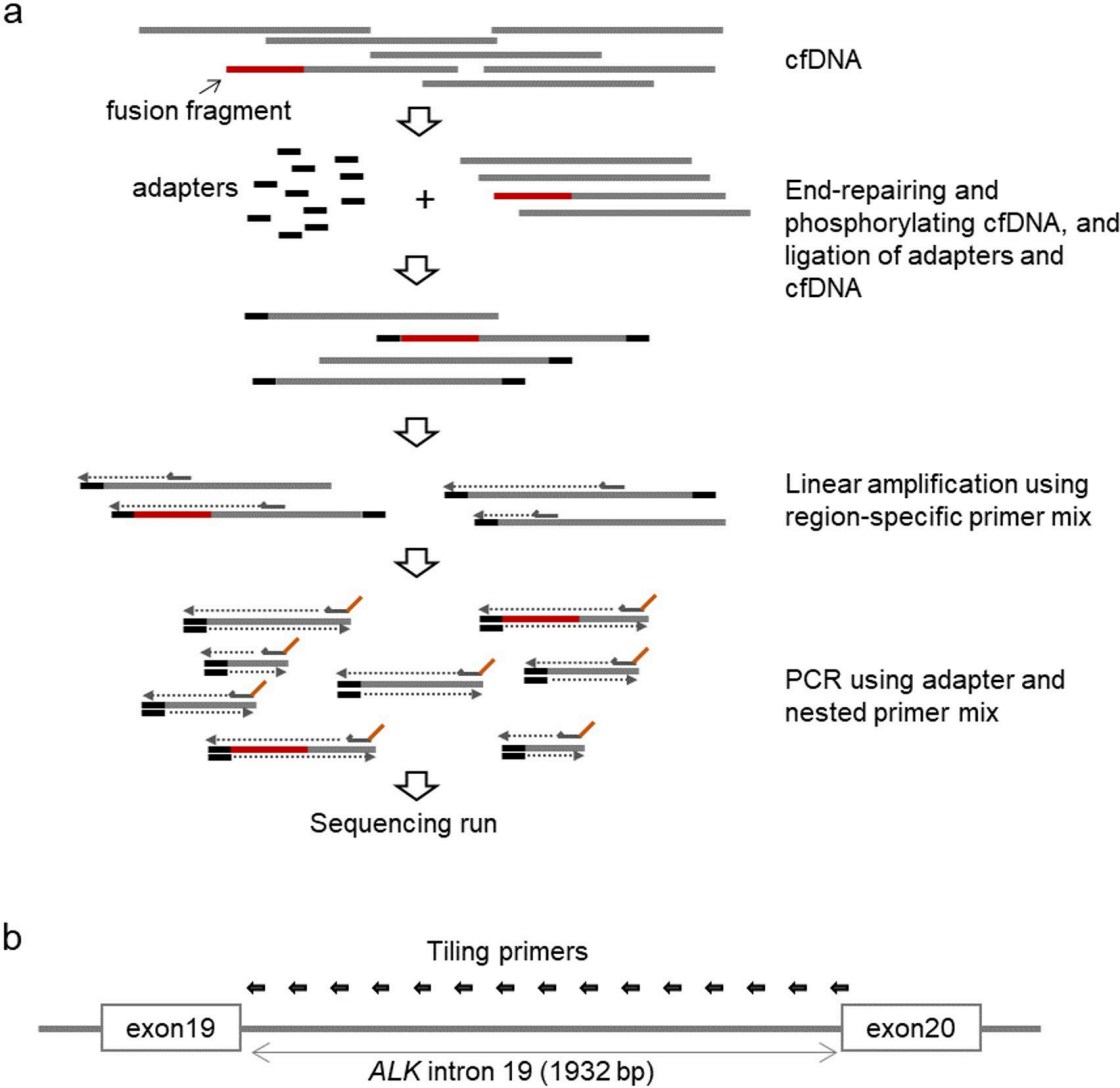
Construction of the sequencing library. a) Cell-free DNA was end-repaired, phosphorylated, and ligated to adapters. DNA fragments with adapters were subjected to linear amplification using a region-specific primer mix. To construct sequencing libraries, the products were amplified using primers including sequences indispensable for the Ion Torrent sequencing system. b) Schematic depicting primer design. To cover the entire intron 19 of *ALK*, we designed a set of 42 tiling primers.

Previously, we developed a multiplex PCR method using adapter-primers with molecular barcodes and single gene-specific primers to analyze point mutations in cfDNAs in the plasma of cancer patients [14, 15]. In this study, we modified the method and applied it for the detection of *ALK* fusions in cfDNAs (Fig 1A). We did not use molecular barcodes, because detection of breakpoints does not need the accuracy required for point mutations. *ALK* fusions are formed by chromosomal rearrangements involving genomic regions of *ALK* and partner genes. Because most of the breakpoints in *ALK* are within intron 19 (1932 bp), we designed two primers each at 42 loci of the intron at approximately 50 bp intervals, covering the entire region (Fig 1B and S1 Table). After adapter ligation, linear amplification was performed with outside primers. Then, PCR amplification was performed using the adapter-primer and inside primers. When the adapter attaches to a partner gene of a breakpoint, a fragment including a breakpoint can be amplified.

We then constructed an analysis pipeline to identify fusions. Although many genes with coiled-coil domain are potential partners, 21 genes are recorded as partner genes of *ALK* fusions in the Catalogue of Somatic Mutations in Cancer (COSMIC v.86) database (https://cancer.sanger.ac.uk/cosmic). More than 60% of detected *ALK* fusions are linked to the *EML4* gene. Certain genes are tumor-type specific: *EML4*-*ALK* fusions have been detected in lung cancer samples, and nucleophosmin (*NPM1*)-*ALK* fusions were found in anaplastic large cell lymphoma (ALCL). Regardless of the types of malignancies, all partner genes of *ALK* fusions in the COSMIC database were included in our analysis pipeline (see Methods and S2 Table). Since searching the whole genome for targets would be time-consuming, we created a list of potential fusion partner targets. This list can be altered as and when the mutation database is updated.

**Fig 2:**
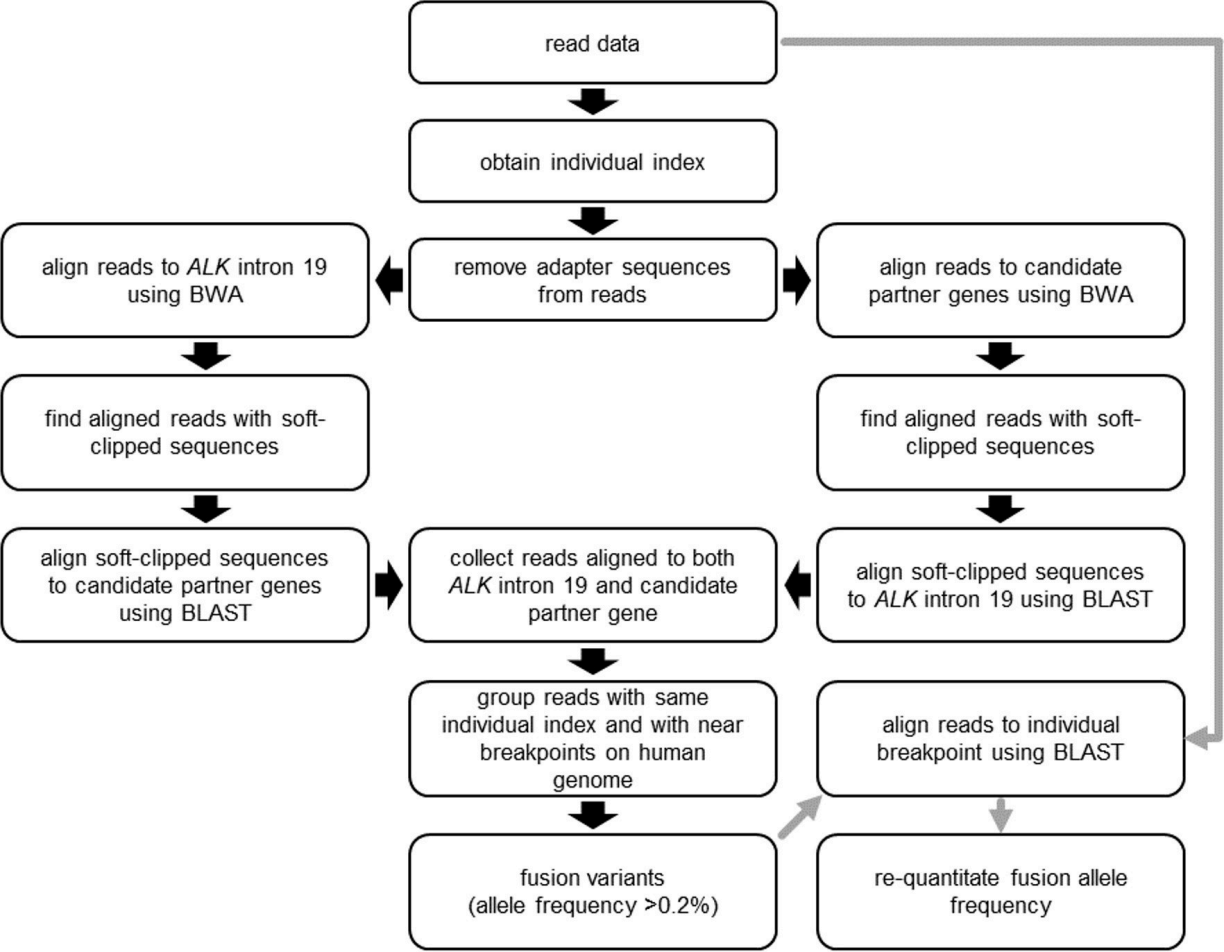
Flowchart of sequencing data analysis. Black and gray arrows denote fusion detection and re-quantification processes, respectively.

As shown in the analysis flowchart (Fig 2), sequencing reads were aligned to *ALK* intron 19 using BWA-MEM [16]. Regarding each aligned read, the unaligned sequence (‘soft-clipped’ sequence) was recovered and re-aligned to exons/introns of the partner gene using BLAST. To increase the ability for detection, alignment was first performed with *ALK* intron 19 and then with those of potential partner genes, and repeated in reverse order. When a read included sequence from both *ALK* intron 19 and a partner gene, we judged that the read was from a fusion. During experiments using dilution series of a reference standard of *EML4-ALK* fusion (see next section), we detected false fusions as well as *EML4-ALK* fusions, and obtained the distribution of their allele fractions (S1 Fig). Allele fraction of the detected false fusions was below 0.05%. Although true *EML4-ALK* fusions were detected above 0.1%, to maximally reduce false positives, we set the threshold of fusion allele detection to 0.2% during the detection process. This is equivalent to three fusion alleles in 5 ng of human genomic DNA (approximately 1500 genome equivalents). In some cases, not all fusion reads may be recovered with the above detection process. Thus, when the fusion allele fraction was over 0.2%, we aligned reads against the 20 bp breakpoint sequence using BLAST and corrected quantitation of fusion alleles, counting those with ≥17 bp matches. A schematic representation of the analysis pipeline is presented in Fig 2.

### Evaluating constructed pipeline for ALK fusion detection

**Fig 3:**
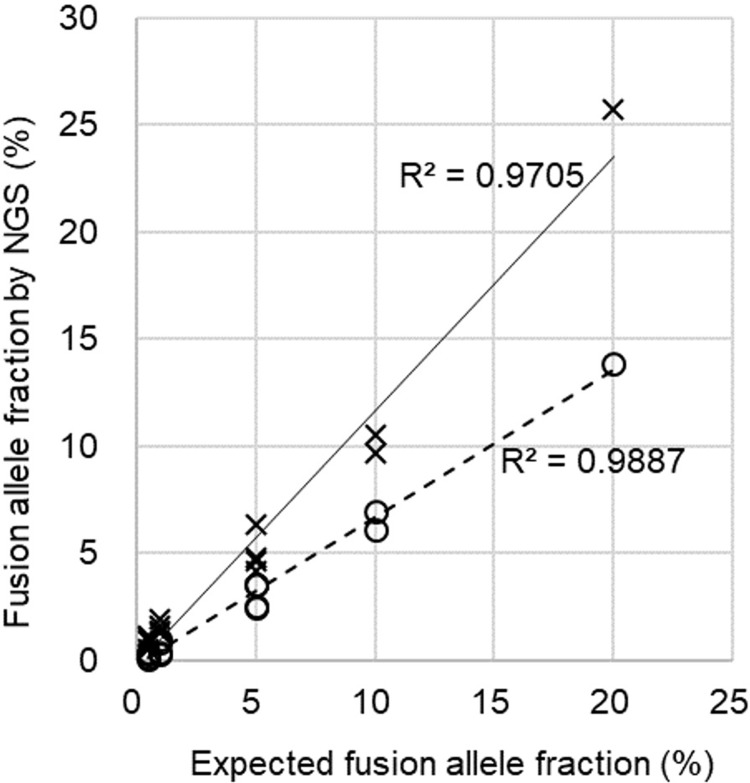
Estimating allele fraction of an artificial fusion by NGS assay. Normal and *EML4*-*ALK* reference standard DNAs were mixed over a range of fusion allele ratios and used as templates for NGS assays. Experiments were performed four times for 0.5, 1, and 5% allele ratios, twice for 10%, and once for 20% ratios. Circles and crosses indicate fusion allele fractions before or after re-quantification of sequencing reads by alignment using 20 bp breakpoint sequences, respectively.

To test our system, we prepared artificial DNAs by combining and fragmenting mixtures of reference standard DNA of *EML4*-*ALK* fusion and normal DNAs, changing the fraction of *EML4*-*ALK* from 0.5 to 20% (see Methods). Using these mixtures, we performed sequencing library construction, DNA sequencing, and fusion detection. Artificial fusion alleles were detected for all sequencing libraries other than for two 0.5% mixtures whose fusion allele fractions were actually lower than 0.2% (i.e., less than the threshold value). Although detected fusion allele fractions and inputs were correlated, the fractions were lower than expected when quantitation was performed without re-quantification using 20 bp breakpoint sequences (circled dots in Fig 3 and S3 Table). After re-aligning reads against each 20 bp breakpoint sequence of the fusion and wild-type counterpart using BLAST (gray arrows in Fig 2), fusion allele fractions were closer to the expected values while maintaining sufficient correlation (crosses in Fig 3 and S3 Table).

### Detection of ALK fusions in NSCLC patients

**Fig 4:**
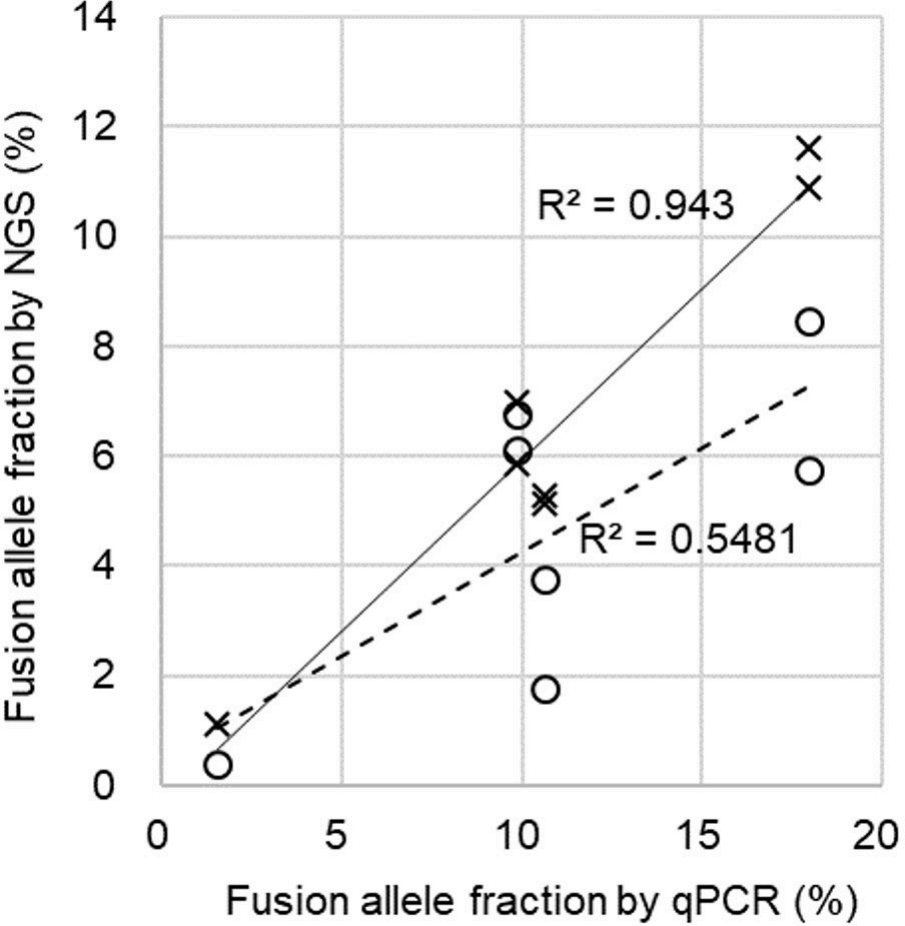
Comparison of fusion allele fractions between NGS assays and qPCR. After re-aligning NGS reads to each breakpoint sequence, quantification of fusion allele fractions using NGS and qPCR were closely correlated (crosses) as opposed to estimates without re-quantification (circles). Data from samples, AK03, AK04, AK07, AK09, and AK10 are plotted. AK03 and AK04 were assayed twice (see Table 1).

We collected 20 blood samples (AK01 to AK20, see Table 1) from NSCLC patients who were diagnosed as *ALK*-positive using FISH, IHC, and/or RT-PCR assays of cancer tissue biopsies. Fifteen samples were obtained before treatment, and five were after treatment initiation with TKIs. As *ALK*-negative controls, we used 10 blood samples from healthy volunteers. cfDNAs were extracted from plasma and at least 10 ng was used for sequencing library construction. After DNA sequencing, reads were analyzed for *ALK* fusions using our pipeline. Each nucleotide position of *ALK* intron 19 was sequenced to more than 1000-fold depth (median depth: 7926 to 24481, S2 Fig and S4 Table). We detected *EML4*-*ALK* fusions in >0.2% fusion allele fractions from 10 blood samples (Table 1). No fusions were detected in healthy controls. Of them, five breakpoints identified using cfDNAs were compared with quantitative PCR (qPCR) assays using breakpoint-specific primers (see Methods). Although correlation of fusion allele fractions was low before re-aligning sequencing reads against individual breakpoint sequences (R^2^ = 0.55, circles in Fig 4), after re-alignment, correlation was dramatically improved (R^2^ = 0.94, crosses in Fig 4). For three patients, bloods were collected again after the appearance of resistance to TKI-therapy: the same breakpoints were detected as in the first assays (Table 1).

**Table 1:** Breakpoints of fusion alleles detected from ALK-positive NSCLC patients.

Sample^a^	*ALK* breakpoint (chr2)	*EML4* breakpoint (chr2)	Breakpoint region of *EML4*	Breakpoint sequence (partner/*ALK*)^b^	FAF1 (%)^c^	FAF2 (%)^c^	Used cfDNA (ng) for NGS	cfDNA (ng) in 10 mL of blood	Therapy	Re-diagnosis^d^
AK01	nd	nd	nd		nd	Nd	10.0	86.5	-	
AK02	nd	nd	nd		nd	Nd	10.0	56.5	-	
AK03	29448042	42523384	intron 13	GTTCTAAAAC/ GATGGTGAAA	5.74	11.60	61.0	955.0	-	
AK03_2	29448042	42523384	intron 13	GTTCTAAAAC/ GATGGTGAAA	8.45	10.89	10.0	955.0	-	
AK04	29447392	42523030	intron 13	AGAGAAAAGG/ GAGTTTGCCT	6.76	6.96	46.0	1295.0	-	
AK04_2	29447392	42523030	intron 13	AGAGAAAAGG/ GAGTTTGCCT	6.13	5.85	10.0	1295.0	-	
AK05	nd	nd	nd		nd	nd	10.0	56.0	-	
AK06	nd	nd	nd		nd	nd	10.0	61.5	-	
AK07	29447000	42504203	intron 6	TTTTTTTCTT/ CTGTGATTGC	1.77	5.28	21.8	238.0	-	
AK08	nd	nd	nd		nd	nd	10.0	50.5	-	
AK09	29447464	42526669	intron 13	TTAGT/tttgg/ CCATGTGTTG	0.41	1.14	20.2	207.0	-	
AK10	29446425	42503561	intron 6	TACTCTCCCA/ GGCCATGTTG	3.75	5.13	10.0	113.0	-	
AK11	nd	nd	nd		nd	nd	10.0	76.8	-	
AK12	29447108	42523339	intron 13	ACTTCCTTCA/ T*AGAGATCTT	0.24	0.32	10.0	47.5	+	
AK13	29446425	42503561	intron 6	TACTCTCCCA/ GGCCATGTTG	0.22	0.41	10.0	111.4	+	same patient as AK10
AK14	29448266	42525419	intron 13		1.95	3.02	10.0	57.0	-	
AK15	29447108	42523339	intron 13	ACTTCCTTCA/ T*AGAGATCTT	0.41	1.25	10.0	75.5	+	same patient as AK12
AK16	nd	nd	nd		nd	nd	10.0	32.4	+	same patient as AK11
AK17	nd	nd	nd		nd	nd	10.0	93.4	-	
AK18	nd	nd	nd		nd	nd	10.0	55.5	-	
AK19	nd	nd	nd		nd	nd	10.0	51.5	-	
AK20	29446425	42503561	intron 6	TACTCTCCCA/ GGCCATGTTG	0.59	0.89	10.0	111.3	+	same patient as AK10

nd: not detected.

^a^AK03 and AK04 were assayed twice using different amount of cfDNA.

^b^An insertion in breakpoint of AK09 is showed in lowercase letters. Asterisked bases of AK12 and AK15 are alternative bases of SNP rs4387740.

^c^FAF1 and FAF2 are fusion allele fractions estimated before or after re-aligning reads using 20-nt breakpoint sequences, respectively.

^d^Three patients were collected bloods during therapy.

Regarding the amount of extracted cfDNAs, >100 ng of cfDNAs per 10 mL blood was extracted from samples with detected fusions (Table 1). In contrast, all samples where fusions were not detected had less than 100 ng of cfDNAs per 10 mL of blood. Hence, the concentration of cfDNA may be important for fusion detection in blood.

There are several reports which described analysis of circulating cell-free RNAs (cfRNAs) and platelet RNAs [17, 18]. For two fusion-positive samples with high fusion allele fractions (AK03 and AK04), we performed RT-PCR of platelet RNA using primers for *EML4* exon 13 and *ALK* exon 20, but no amplicons were observed. Since RNA is generally less stable than DNA, it might have undergone degradation when the platelet samples were frozen. Assays of cfRNAs were not examined any more as they were outside the scope of our study.

### Characterization and distribution of ALK breakpoints in NSCLC patients

We identified seven breakpoints, with one (AK09) containing a 5 bp insertion at the fusion breakpoint. No apparently homologous sequences were found around the breakpoints (Tables 1 and S5). The same characteristic has been observed in breakpoint regions of *EML4*-*ALK* fusions detected by other groups (S5 Table) [3, 9, 11, 12, 19]. These observations suggested that *EML4*-*ALK* fusions in NSCLC patients were formed via non-homologous end joining repair as *NPM1*-*ALK* fusions in ALCL patients [20].

**Fig 5:**
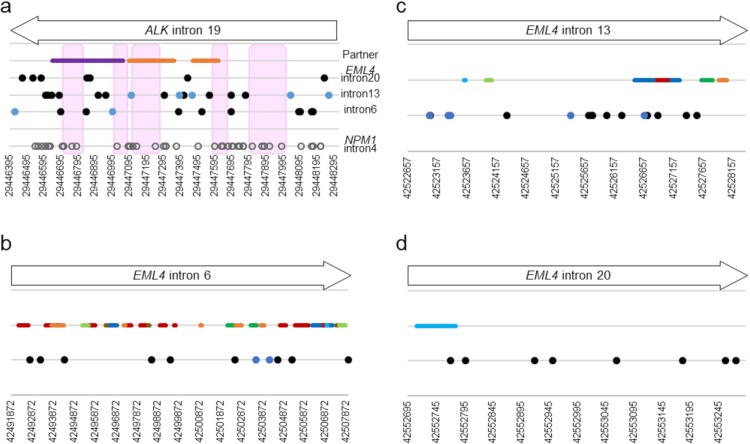
Graphic representation of *ALK* fusion breakpoints. a) *ALK* intron 19, b) *EML4* intron 6, c) intron 13, and d) intron 20 are shown. Regions where breakpoints are absent are shaded pink. Breakpoints of fusions are denoted by black (reported in previous studies), blue (detected in present study), and white circles (reported in ALCL study). Colored lines indicate repetitive elements, *Alu* (red), hAT-Charlie DNA transposon (green), L1/L2 (blue), AT-rich (brown), MIR (Mammalian-wide Interspersed Repeat) (orange), simple repeat (black), TcMar-Tigger DNA transposon (light green), and LTR (Long Terminal Repeat) (purple).

Breakpoints within *ALK* intron 19 in NSCLC patients, including those previously reported, are shown in Fig 5A and S5 Table [3, 9, 11, 12, 19]. In intron 19, there are three repetitive elements; a long terminal repeat retrotransposon LTR16B2 and two mammalian-wide interspersed repeats (MIRc and MIR). These repetitive element families occupy approximately 10% of the human genome [21] and may mediate genomic rearrangements. There are five regions where breakpoints are absent (size range, 108 to 264 bp) (Fig 5A): of these, four overlapped with repetitive elements and one was outside the repetitive elements. Breakpoints of *NPM1*-*ALK* fusions in ALCL patients were distributed throughout *ALK* intron 19 without any significant correlation with repetitive elements [20]. In both cases, breakpoints are not likely to be associated with the repetitive elements. There was also no apparent association between breakpoints and repetitive elements in introns of *EML4* (Fig 5B, 5C and 5D).

## Discussion

In this study, we first devised a PCR-based target sequencing system using an adapter and a tiling primer set that cover the entire intron 19 of *ALK*. Because primers should be designed at short intervals (approximately 50 bp) to accommodate the small size of cfDNA, flexibility in primer design is restricted and may increase artifactual amplification products. In addition, our PCR system, using a common adapter-primer and single gene-specific primers, may also lead to the same problem. However, this issue can be overcome by increasing the number of sequencing reads. The depth of our sequencing was at least 1000-fold (S2 Fig and S4 Table), ensuring sensitivity of fusion detection. Hybridization-capture methods employ 3-5-fold longer probes than ours: a previous study pointed out inefficient recovery of *ALK* fusion breakpoints with longer probes using cfDNA [12].

Secondly, we constructed an analysis pipeline for quantitative detection of *ALK* fusions. Because breakpoints of fusions consist of two different sequences in short cfDNA fragments, fusion detection and quantification are affected by sequences surrounding breakpoints. Some breakpoints contain small insertions or SNPs that hamper alignment-based detection of fusions, and a considerable fraction of reads, including breakpoints, may escape matching. In this study, we employed a two-step strategy that firstly detects fusions by sequencing with adapter-PCR-based library construction and read-alignment, and, secondly, quantitates detected fusions by aligning them to their own short breakpoint-sequences. This second step is important for accurate quantification of breakpoints, especially when the breakpoints include insertions or SNPs. It should be noted that the Ion Torrent sequencing system generates more indel errors than Illumina platforms [22, 23]. Quantification of ctDNA in blood from cancer patients has been used for measuring the effects of therapy and monitoring tumor burden during treatment, indicating potential clinical merit of our technical approach. We would stress that our quantification method is applicable to short reads on any sequencing platform.

Wang et al [11] examined 37 blood samples from 24 NSCLC patients with confirmed *ALK* rearrangements based on their tissue biopsies using hybridization capture-based sequencing. They detected *ALK* fusions in 28 bloods with 76% sensitivity and 100% specificity (from 36 *ALK* rearrangement negative cases in tissue biopsies). Their result is better than the results of our study (50% sensitivity and 100% specificity), although the two studies cannot be directly compared because the number of patients and their conditions were not the same. The commercialized targeted-sequencing panel used in Wang et al’s study targeted 168 genes including *ALK* and spanned 160 kb genomic regions. The targeted-capture method used in this panel is designed for large genomic regions; hence, costly sequencing is required excessively to obtain reliable read depth for each target even after including unnecessary regions in the target sequencing panel. Additionally, such methods are difficult to scale to small targets because capturing-efficiency of targets is too low (2–5%) unless an additional laborious round of PCR and capture process is added [24]. Thus, our method can be used as an alternate approach for *ALK* fusion detection because it is more economical than the typical targeted capture-based sequencing when rearrangements are the only targets.

In the present study, we detected fusion breakpoints in 10 of 20 blood samples from *ALK*-positive patients and not in negative controls (healthy volunteers). The amount of ctDNA is diverse among patients, with a tendency that more ctDNA can be detected in patients with malignant tumors [25]. As described in the Results section, NSCLC patients with detected fusions had more abundant cfDNA (>100 ng cfDNA per 10 mL of blood) than in patients where fusions were not identified, although there were also exceptions. A recent report also showed a weak positive correlation between the variant allele fraction of driver genes and cfDNA concentration in NSCLC patients [26]. Owing to the small sample size of our study, we need to confirm the relationship between cfDNA and ctDNA through experiments with strictly controlled parameters, such as amount of blood and cfDNA of samples for assay. Additionally, for rare fusion fragments, increasing the amount of plasma for assays may improve fusion detection. Moreover, because all cfDNA fragments are not captured during library preparation, further refinement of library preparation (especially adapter-ligation step) may lead to detection of more fusions from patients with relatively low fusion allele fraction.

## Conclusions

We combined PCR-based target sequencing and mapping/alignment programs that are specific to quantitative detection of *ALK* fusions in cfDNA. The performance indicates that our method is a viable option for molecular diagnosis of *ALK* status in NSCLC patients using cfDNA.

## Materials and methods

### Plasma cfDNAs

*ALK*-positive lung cancer patients and healthy volunteers were recruited, and blood samples were obtained between September 2017 and February 2019 at the Osaka International Cancer Institute, Japan. Their tumor tissues were diagnosed for *ALK* status using IHC, FISH and/or RT-PCR. Ten milliliters of blood were collected with a Cell-Free DNA BCT whole blood collection tube (Streck, La Vista, NE, USA) and sent to Nara Institute of Science and Technology, where cfDNA samples were analyzed. Within 2 days, plasma was separated via centrifugation of collected blood as per the manufacturer’s instructions. Plasma was centrifuged again at 15 100 × *g* for 10 min to remove cellular debris and stored at -80°C until extraction of cfDNA. This study was approved by the ethics committees of the Osaka International Cancer Institute and Nara Institute of Science and Technology. Written informed consent was obtained from all participants recruited for this study.

cfDNA was extracted using the QIAamp Circular Nucleic Acid Kit (Qiagen, Hilden, Germany) according to the manufacturer’s instructions. DNA concentrations were measured using Qubit dsDNA HS Assay Kit (Thermo Fisher Scientific, Waltham, MA, USA). 32–1295 ng of cfDNA per blood sample was obtained.

### Reference standard EML4-ALK fusion DNA

We used EML4-ALK Reference Standard (Horizon Diagnostics, Cambridge, UK) as a DNA reference standard for *EML4*-*ALK* gene fusion. Whole genome amplification of EML4-ALK Reference Standard was performed using GenomiPhi V2 (GE Healthcare, Chicago, IL, USA). After purification using Microcon 100 (Merck Millipore, Burlington, MA, USA) centrifugation filters, amplified DNA was mixed with pooled healthy volunteer DNAs (Megapool Reference DNA (male); Leica Biosystems, Wetzlar, Germany) in the ratio of 2:3 to finally represent 20% of *EML4*-*ALK* fusion allele content. The mixture was fragmented with Fragmentase (NEB, Ipswich, MA, USA). To obtain DNA fragments similar to the lengths of cfDNA, fragmented DNA was treated using AMPureXP (Beckman Coulter, Brea, CA, USA) double size selection (using 0.8× AMPureXP for the first selection and 0.7× for the second). DNA solutions with various proportions of *EML4*-*ALK* fusion alleles were made by combining the above fragmented DNA and fragmented Megapool DNA.

### Construction of NGS library and DNA sequencing

We constructed NGS libraries by employing similar procedures as described in a previous study (Fig 1) [14]. All oligonucleotides used are listed in S1 Table. NGS is known to be more error-prone than conventional Sanger sequencing. As one of the solutions to improve the accuracy of determining DNA sequences, molecular barcoding technologies have been developed and are currently prevalent in mutation detection. However, they require a large number of sequencing reads to construct a consensus of sequences with the same barcodes [14, 15]. For fusion detection, high accuracy is not needed because the fusion alleles result after megabase-level changes to chromosomes, which is different from single nucleotide variants (SNVs). Thus, although we used adapters with molecular barcodes in previous studies [14, 15], the barcode information was not analyzed in this study to reduce production of sequencing reads. cfDNA was end-repaired in 15–45 μL of reaction solution (50 mM Tris-HCl, pH 8.0, 10 mM MgCl_2_, 10 mM dithiothreitol, 1 mM ATP, 0.4 mM dNTPs, 0.16 units/μL of T4 DNA polymerase (Takara Bio, Shiga, Japan), 0.5 units/μL of T4 polynucleotide kinase (NEB) and 0.03 units/μL of KOD DNA polymerase (Toyobo, Osaka, Japan)) with incubation of 30 min at 25°C and 20 min at 75°C. The ligation of adapters was performed in 20–60 μL of reaction solution (the end-repair solution, 1/40 volume of 10× T4 DNA ligase buffer (NEB), 2 μM of adapter, 100 units/μL of T4 DNA ligase (NEB)) at 25°C for 15 min. The ligation products were purified twice with a 1.2× volume of AMPureXP beads. The purified beads were dissolved in 20 μL of the linear amplification solution (1× High Fidelity PCR Buffer (Thermo Fisher Scientific), 0.2 mM dNTPs, 2 mM MgSO_4_, 6 μM region-specific primer mixO (S1 Table), and 1 unit of Platinum Taq DNA High-Fidelity Polymerase (Thermo Fisher Scientific)). After removal of the AMPureXP beads, amplification was performed as follows: 2 min at 95°C for denaturation and 15 cycles of 15 sec at 95°C, 2 min at 65°C. Then, 1.2 μL of 100 μM T_PCR_A primer was added to the reaction, then amplified by 15 cycles of 15 sec at 95°C, 30 sec at 65°C, and 30 sec at 72°C. The amplification products were purified with a 1.2× volume of AMPureXP and recovered in 20 μL of 0.1× TE. Purified products (2 μL each) were divided into eight tubes for nested PCR. For eight nested primer mixes (A~H, S1 Table), the purified products were re-amplified in 20 μL of PCR solution (1× High Fidelity PCR Buffer (Thermo Fisher Scientific), 0.2 mM dNTPs, 2 mM MgSO_4_, 0.5 μM T_PCR_A, 0.5 μM nested primer mix, and 0.4 units of Platinum Taq DNA High Fidelity Polymerase (Thermo Fisher Scientific)). Thermal cycling was performed as follows: 2 min at 95°C for denaturation and 30 cycles of 15 sec at 95°C and 1 min at 63°C. The amplification products were purified with a 1.2× volume of AMPureXP beads. The concentration was determined using the Qubit dsDNA HS Assay Kit or Quant-iT PicoGreen dsDNA Assay Kit (Thermo Fisher Scientific). NGS libraries from 4–8 blood samples were combined and used for sequencing by an Ion PGM sequencing system with Hi-Q View reagents and Ion 318 Chips (Thermo Fisher Scientific). After sequencing runs, FASTQ files of sequencing data were extracted using Torrent Suite (Thermo Fisher Scientific).

### Target fusion partner regions

We extracted partner genes of *ALK* fusions recorded in COSMIC v86 and targeted exons/introns with inferred breakpoints. They were as follows, *ATIC* exon/intron 7, *C2orf44* exon 4, *CARS* exon/intron 17, *CLTC* exons/introns 30, 31, *DCTN1* exons/introns 16, 26, *EML4* exons/introns 2, 6, 13, 14, 15, 17, 18, 20, *FN1* exon/intron 23, *HIP1* exons/introns 21, 28, 30, *KIF5B* exons/introns 15, 17, 24, *KLC1* exon/intron 10, *MSN* exon/intron 11, *NPM1* exon/intron 5, *PPFIBP1* exons/introns 8, 12, *RANBP2* exon/intron 18, *SEC31A* exon/intron 20, *SQSTM1* exon/intron 5, *STRN* exon/intron 3, *TFG* exons/introns 4, 5, 6, *TPM3* exon/intron 7, *TPM4* exon/intron 7, and *VCL* exon/intron 16. Details regarding nucleotide positions are shown in S2 Table.

### Detection and quantification of fusion alleles involving ALK and partner loci

Sequencing reads in FASTQ files were analyzed in accordance with the flowchart presented in Fig 2. As shown at left in Fig 2, reads with removed adapter sequences were mapped onto *ALK* intron 19 and parts of exon 19 and 20 (chr2:29446370–29448369 on GRCh37/hg19) using the BWA-MEM mapping program [16]. Reads with soft-clipped sequences were collected, and these sequences were aligned against candidate partner sequences using the BLAST alignment program [27]. At the right in Fig 2, reads were mapped onto candidate fusion partner sequences, and soft-clipped sequences were aligned against *ALK* intron 19 sequence. Then, reads mapped/aligned on both *ALK* intron 19 and candidate partner sequences were collected and grouped using information of individual index sequences and breakpoint positions. These processes were performed using in-house Perl scripts. Fusion allele fractions were calculated as follows; counts of reads with fusion alleles/sequencing depth of *ALK* intron 19 at the breakpoint × 100 (%). When the fusion allele fraction was over 0.2%, we aligned reads against the 20 bp breakpoint sequence using BLAST and corrected quantitation of fusion alleles. The 20 bp breakpoint sequences were prepared by combining 10 bp of the *ALK* side and 10 bp of the partner side around detected breakpoints. We also prepared counterpart sequences for *ALK* wild-type. After raw sequencing reads assorted using individual indexes were aligned against the 20 bp sequences, we collected alignments with ≥17 bp matches and calculated fusion allele fraction as follows; number of alignments of fusions/(number of alignments of fusions + number of alignments of wild-type) × 100 (%).

### Quantitative PCR

Primer sequences are listed in S1 Table. We used 5 ng of cfDNA and a SYBR Green-based reagent kit, TB Green Premix Ex Taq II (Takara Bio). Thermal cycling was performed on a LightCycler 480 system (Roche Molecular Systems) as follows: 30 sec at 95°C for denaturation, then 45 cycles of 5 sec at 95°C and 30 sec at 60°C. For melting curve analysis, 0 sec at 95°C, 15 sec at 65°C, and 0 sec at 95°C were used. We analyzed qPCR data using LinRegPCR [28]. Using estimated starting concentration (N_0_ value), the fusion allele fraction (%) was calculated as follows, N_0_ of amplicon for fusion/(N_0_ of amplicon for fusion + N_0_ of amplicon for wild-type) × 100.

## Supporting information

S1 Fig Histogram of candidate fusions detected from dilution series of mixtures of fusion reference standard and normal DNAs during the detection process. Allele fraction of the detected false fusions was below 0.05%. *EML4-ALK* fusions were detected above 0.1%.
(PDF)

S2 Fig Sequencing depth for intron 19 of *ALK*. X-axis represents the sequence position on chr2. Y-axis denotes the sequencing depth. (a) *ALK*-positive NSCLC patients. (b) Healthy volunteers.
(PDF)

S1 Table List of oligonucleotides. (XLSX)

S2 Table Target fusion partner regions. (XLSX)

S3 Table Fusion allele fractions of artificial DNA mixtures. (XLSX)

S4 Table Statistics of sequencing. (XLSX)

S5 Table Sequences around breakpoints. (XLSX)
