# Supplementary figures and images for: Quantitative detection of ALK fusion breakpoints in plasma cell-free DNA from patients with non-small cell lung cancer using PCR-based target sequencing with a tiling primer set and two-step mapping/alignment
Source: PLoS One. 2019 Sep 12;14(9):e0222233. doi: 10.1371/journal.pone.0222233 (PMC6742348; doi:10.1371/journal.pone.0222233)

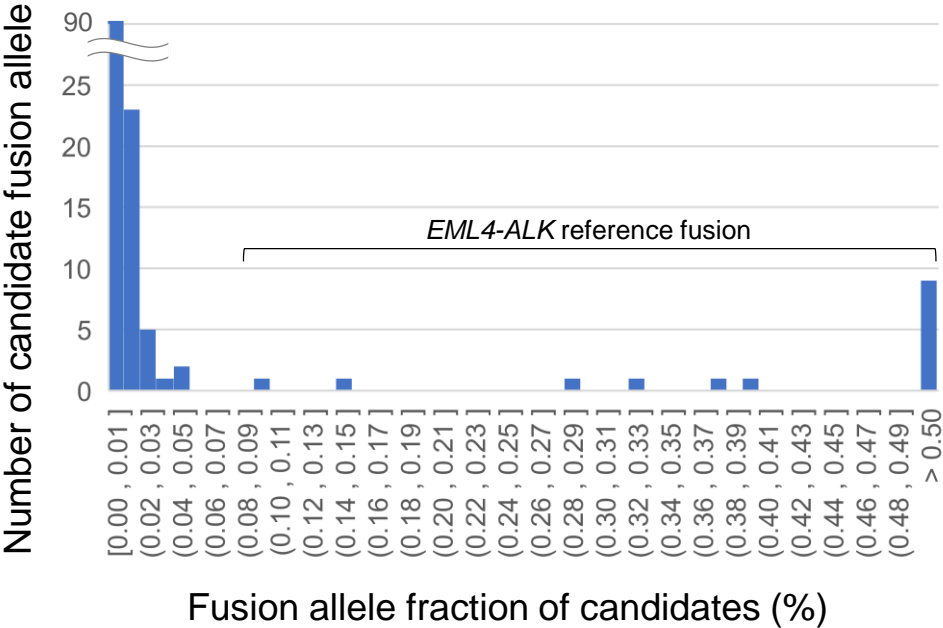

Supplement: S1 Fig — Allele fraction of the detected false fusions was below 0.05%. EML4-ALK fusions were detected above 0.1%. (PDF) [file pone.0222233.s001.pdf]

S2a Fig

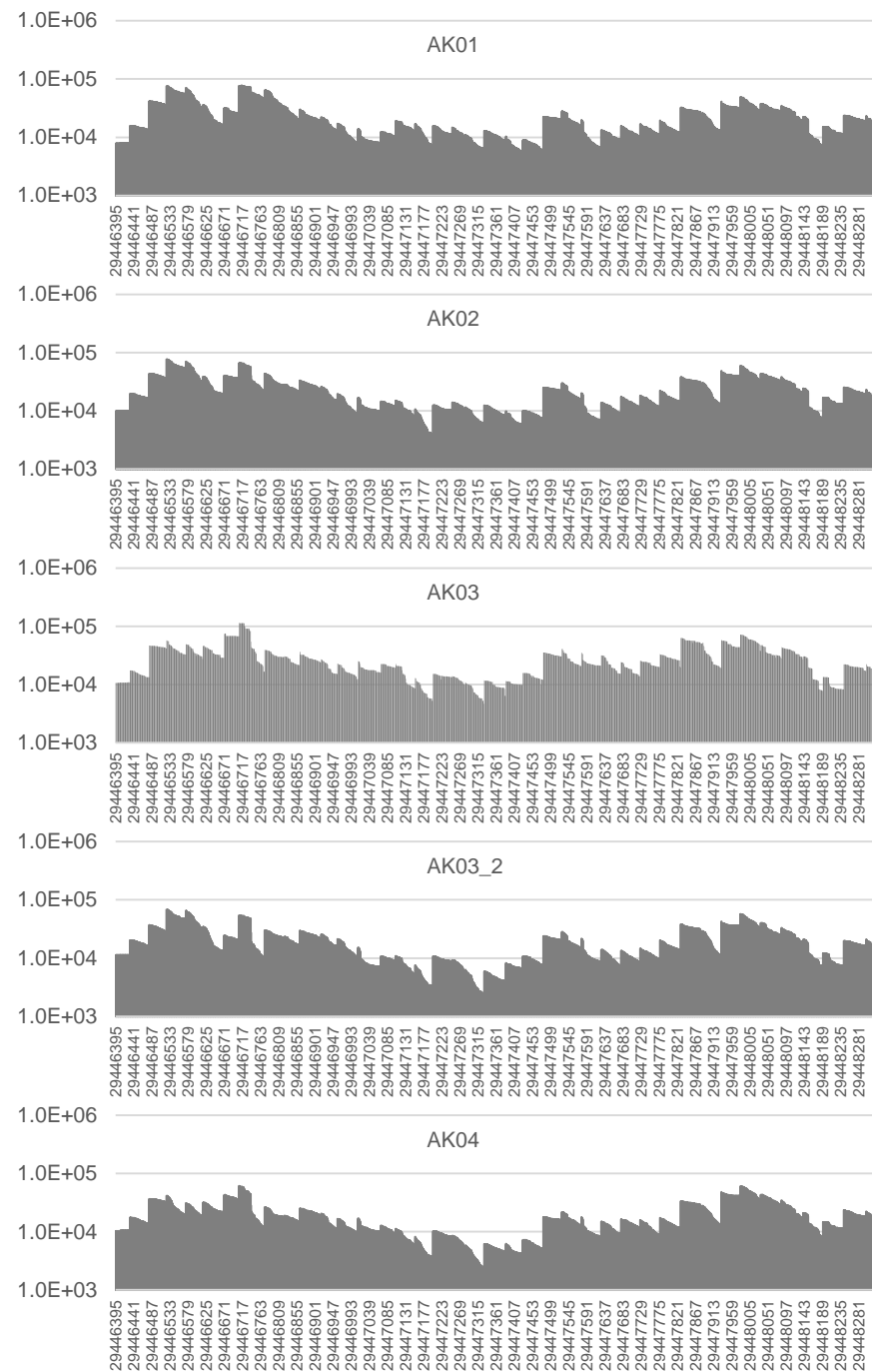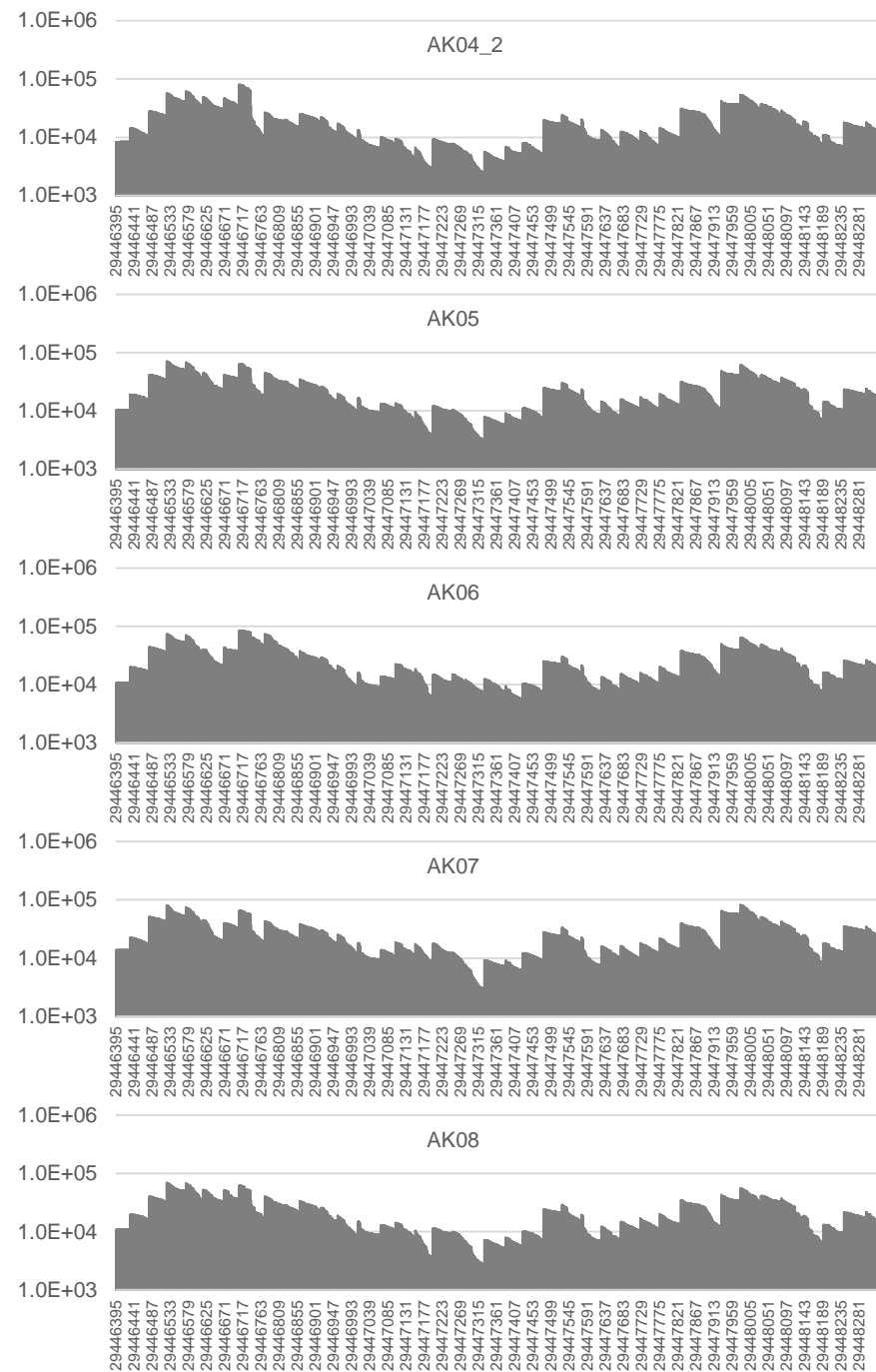

S2a Fig. (continued)

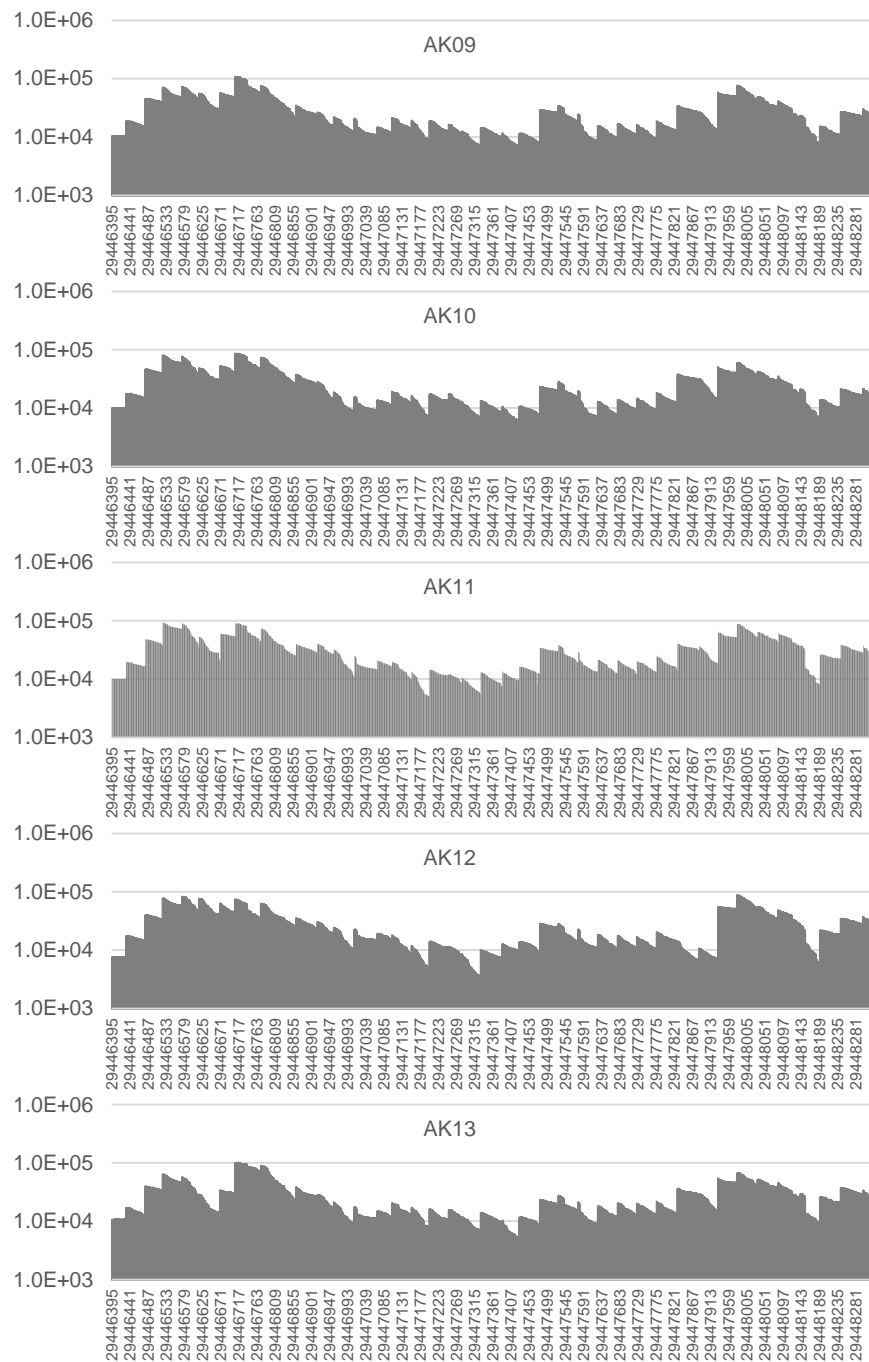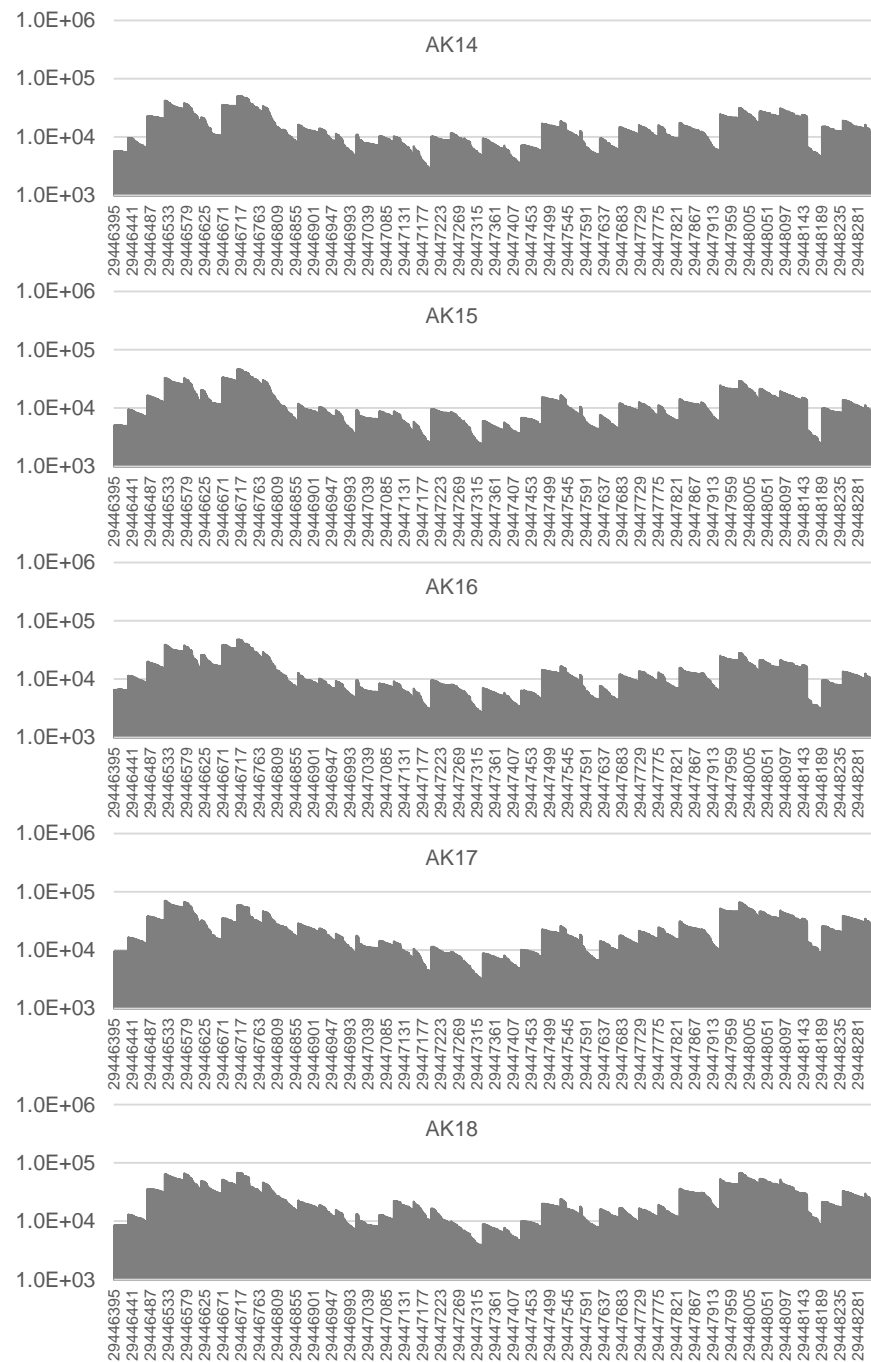

S2a Fig. (continued)

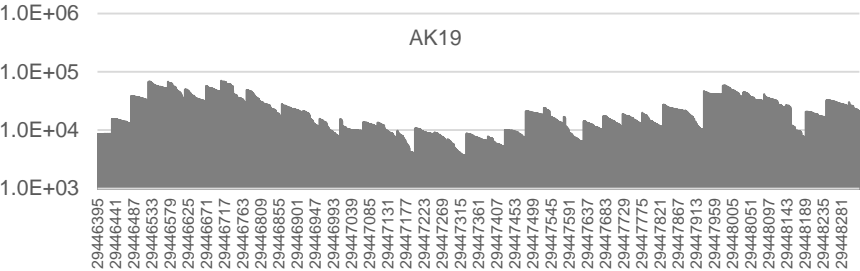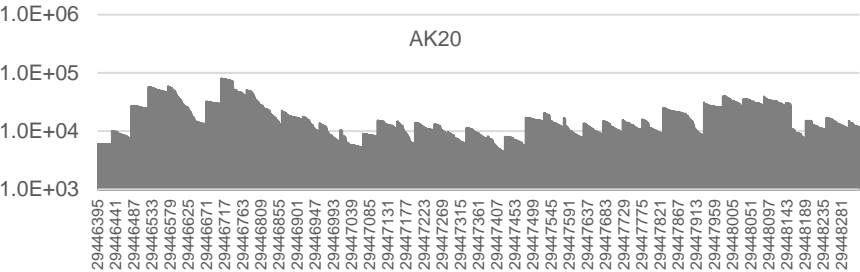

S2b Fig.

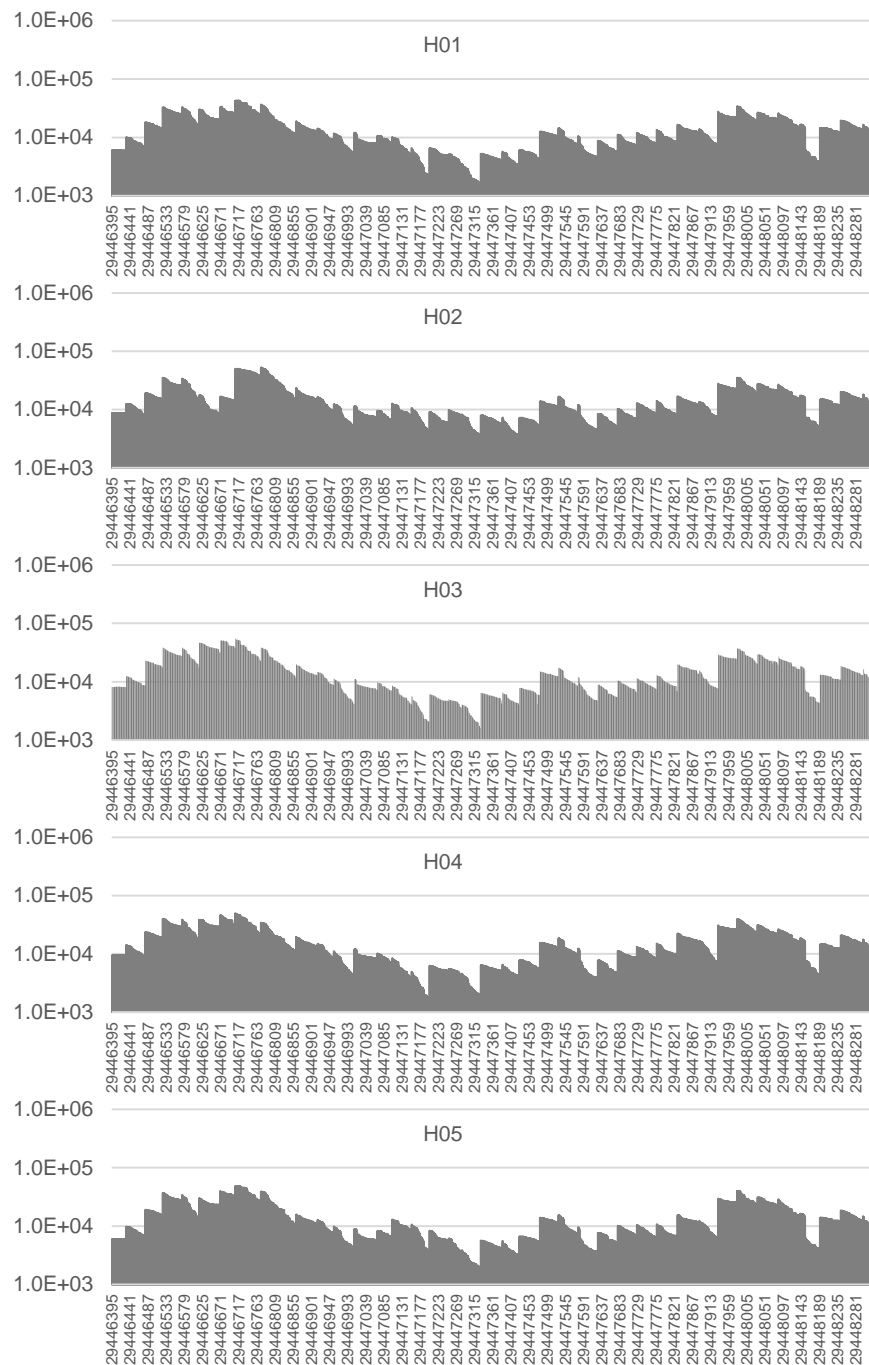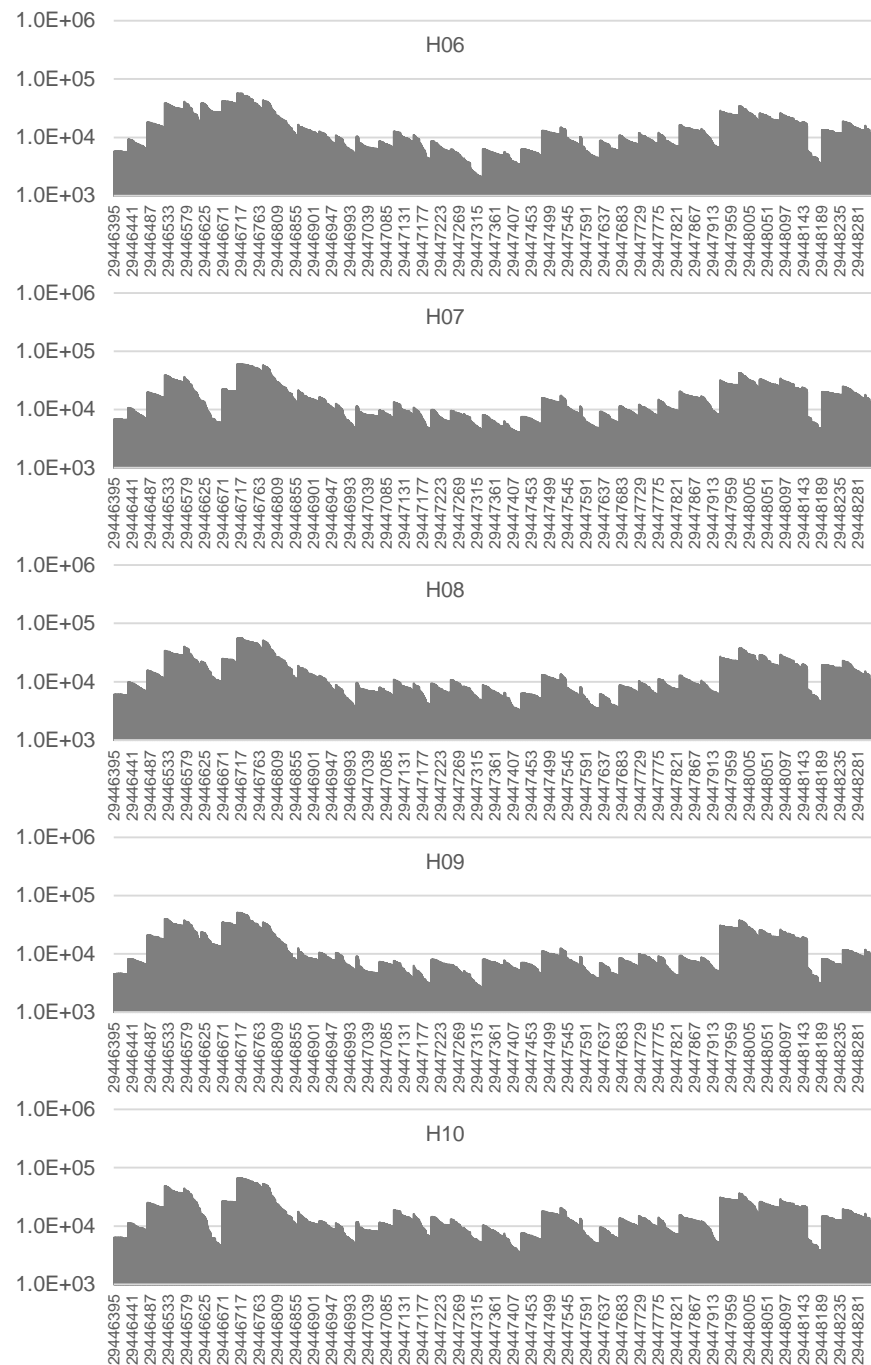

Supplement: S2 Fig — X-axis represents the sequence position on chr2. Y-axis denotes the sequencing depth. (a) ALK-positive NSCLC patients. (b) Healthy volunteers. (PDF) [file pone.0222233.s002.pdf]
